# O095: Isolation videoclip for visitors and patients

**DOI:** 10.1186/2047-2994-2-S1-O95

**Published:** 2013-06-20

**Authors:** BS Ana, CA Palos

**Affiliations:** 1 Infection Control Comittee, Hospital Beatriz Ângelo, Loures, Portugal

## 

HAI prevention and control applies to all settings of care. Screening and isolation of patients are tools that improve HAI prevention and control effectiveness. Awareness, understanding and compliance to different isolation precautions are a major problem for patients and visitors. This videoclip aims to introduce visitors and patients on the hospital isolation policy and procedures, explaining them in a simple and attractive way how to proceed, using a combination of avatar technology and real persons. Videoclips are a part of a global awareness and teaching materials on Infection Control and Prevention.

## Disclosure of interest

None declared.

